# HemoKinect: A Microsoft Kinect V2 Based Exergaming Software to Supervise Physical Exercise of Patients with Hemophilia

**DOI:** 10.3390/s18082439

**Published:** 2018-07-26

**Authors:** Fernando Mateo, Emilio Soria-Olivas, Juan J. Carrasco, Santiago Bonanad, Felipe Querol, Sofía Pérez-Alenda

**Affiliations:** 1 Department of Electronic Engineering, University of Valencia, Avda. Universitat, 46100-Burjassot, Spain; fernando.mateo@uv.es (F.M.); emilio.soria@uv.es (E.S.-O.); 2 Intelligent Data Analysis Laboratory, University of Valencia, Avda. Universitat, 46100-Burjassot, Spain; 3 Department of Physiotherapy, University of Valencia, Carrer de Gascó Oliag, 5, 46010-Valencia, Spain; felipe.querol@uv.es (F.Q.); Sofia.Perez-Alenda@uv.es (S.P.-A.); 4 Haemostasis and Thrombosis Unit, University and Polytechnic Hospital La Fe, Avinguda de Fernando Abril Martorell, 106, 46026-Valencia, Spain; bonanad_san@gva.es

**Keywords:** hemophilia, Kinect, physical exercise, exergaming, rehabilitation

## Abstract

Patients with hemophilia need to strictly follow exercise routines to minimize their risk of suffering bleeding in joints, known as hemarthrosis. This paper introduces and validates a new exergaming software tool called HemoKinect that intends to keep track of exercises using Microsoft Kinect V2’s body tracking capabilities. The software has been developed in C++ and MATLAB. The Kinect SDK V2.0 libraries have been used to obtain 3D joint positions from the Kinect color and depth sensors. Performing angle calculations and center-of-mass (COM) estimations using these joint positions, HemoKinect can evaluate the following exercises: elbow flexion/extension, knee flexion/extension (squat), step climb (ankle exercise) and multi-directional balance based on COM. The software generates reports and progress graphs and is able to directly send the results to the physician via email. Exercises have been validated with 10 controls and eight patients. HemoKinect successfully registered elbow and knee exercises, while displaying real-time joint angle measurements. Additionally, steps were successfully counted in up to 78% of the cases. Regarding balance, differences were found in the scores according to the difficulty level and direction. HemoKinect supposes a significant leap forward in terms of exergaming applicability to rehabilitation of patients with hemophilia, allowing remote supervision.

## 1. Introduction

Hemophilia A and B are hereditary disorders characterized by a deficiency in clotting factors VIII and IX, respectively. This deficiency causes a chronic tendency to hemorrhage [1]. The frequency of this disease is low, so hemophilia is known as a rare disease; for example, hemophilia A occurs in about one in every 5000–6000 live newborns and hemophilia B in one in 30,000 [2]. However, despite the low incidence and prevalence, the cost associated with this disease is one of the most elevated, so it has a great impact on the health system [3]. One of the most important and most increased morbidities in patients with hemophilia is bleeding occurrence in joints, known as hemarthrosis [4]. This bleeding may occur due to a blow or spontaneously, due to friction during the natural movement of the joint. Without proper treatment, recurrent hemarthrosis causes hemophilic arthropathy [5], which involves chronic pain and functional disability of the joint [6]. In patients with hemophilia, bleeding occurs mostly in the knee (44%), elbow (25%), ankle (15%), shoulder (8%), hip (5%) and in other locations (3%).

Health benefits that involve physical activity for the general population are equally applicable to people with hemophilia. An adequate muscle tone avoids injuries and decreases the risk of bleeding in joints. Physical exercise is of great importance for the hemophilic population because it improves their quality of life [7]. There are many studies that address the importance of physical training to improve health condition. They emphasize the World Health Organization recommendations [8] for the general population and World Federation of Hemophilia recommendations for the hemophilic population [7,9,10].

In particular, the use of video games for rehabilitation (exergaming) is having a positive impact on the patients’ attitudes towards training and has proven useful to enhance their strength, coordination and mobility [11,12]. Furthermore, motion capture (MoCap) sensors are being increasingly used for applications in medicine and in physical therapy, as these sensors are becoming readily available in the market and relatively inexpensive against other alternatives such as 3D optical MoCap systems [13].

A popular choice of a MoCap sensor is Kinect, which has been widely (and successfully) used for rehabilitation in a wide range of medical applications [14,15,16,17,18], such as post-stroke limb rehabilitation, elderly exercise monitoring and fall prevention [19], range-of-motion (ROM) evaluation in patients with adhesive capsulitis [20], balance and postural control assessment and training [21,22], virtual gyms for people with restricted mobility [23], etc.

The validity of the first version of Kinect (V1) for clinical applications has been the object of study of several publications [22,24,25]. Several studies [26,27] point out the limited accuracy of Kinect V1 against a clinical goniometer for joint angle measurement, although other recent studies have reported excellent agreement between their measurements [20]. Kinect V2 has been reported as more accurate than V1 [28,29,30] and has recently attracted attention to devise and evaluate a variety of exercises aimed at rehabilitation for patients with axial disorders [31], ROM measurements for home rehabilitation [32] and gait parameter measurement [33].

Until now, only two studies have used MoCap techniques in patients with hemophilia: one for rehabilitation [34] and the other for balance assessment [35]. In both studies, a Nintendo Wii Balance Board was used. Therefore, the main objective of this work is to create a redistributable software tool, HemoKinect, that sets up a number of key exercises to be performed in front of Kinect V2. HemoKinect allows patients to perform them interactively either at health facilities or comfortably at their homes through a user-friendly graphical user interface (GUI). The specialist should be able to remotely receive and interpret feedback from the patients’ activity, via comprehensive periodical reports, to keep track of their progress and adjust their training.

## 2. Materials and Methods

### 2.1. Hardware and Software Description

Kinect V2 (Microsoft Corp., Redmond, WA, USA) is a physical device originally intended for video game interaction using body motion for Microsoft X-Box${}^{TM}$ platforms (Microsoft Corp., Redmond, WA, USA). The release of an SDK by Microsoft [36] has given the programmer community a powerful tool to build their own applications and games using standard programming languages. The first version of Kinect (Kinect V1, Microsoft Corp., Redmond, WA, USA) had some limitations in terms of resolution, accuracy, body tracking and robustness in changing lighting conditions that were greatly overcome by V2 [37]. The most interesting feature of Kinect V2 for our purposes is skeletal tracking. It is possible to retrieve the skeletal data of up to six users from the captured depth maps using machine learning algorithms [38]. The skeleton data for one body is composed of 25 joints that are joined by segments. When a joint is not visible (i.e., hidden behind some furniture), its position may be inferred by the sensor.

HemoKinect has been developed partially in C++ and in MATLAB (MATLAB R2016a, The MathWorks Inc., Natick, MA, USA) [39], using the MATLAB compiler to generate the installer files. It is a standalone application that presents a user-friendly GUI developed with MATLAB GUIDE (GUI development environment). The compiled application is bundled with all the necessary MATLAB routines to execute HemoKinect in any Windows PC without the need for a MATLAB installation or license. HemoKinect software is publicly available for users via GitHub at https://github.com/fermaji/HemoKinect.

### 2.2. Measurement and Methodology

The Kinect V2 was placed at *y* = 1.2 m above the ground using a dedicated floor stand, within Microsoft’s recommended range (0.6 m–1.8 m). The subjects to monitor stood at a longitudinal distance *z* = 2.5 m from the sensor, also within the recommended range (0.5 m ≤z≤ 4.5 m). The exercises were performed by subjects in a standing position, with light clothes to avoid hindering Kinect’s joint center identification, and facing the sensor to optimize joint center identification [40]. To avoid possible loss of accuracy due to joint occlusion, patients were asked to perform all the exercises facing the sensor to keep all joints of interest within its line of sight.

HemoKinect relies on the accuracy of Microsoft Kinect SDK v2.0 libraries to obtain 3D joint positions. This skeletal data were extracted from the depth images using a random decision forest algorithm [38]. After the validation of the captured frames, the angle associated with each joint of interest was calculated by defining a pair of vectors $\left(\vec{u},\vec{v}\right)\in R^{3}$ formed by the joint’s adjacent body segments and obtaining the angle α in degrees between them, as indicated by Equation (1).

(1) $$\alpha =\frac{180}{\pi }\arctan\frac{|\vec{u}\times \vec{v}|}{\vec{u}\cdot \vec{v}}$$

**Figure 1:**
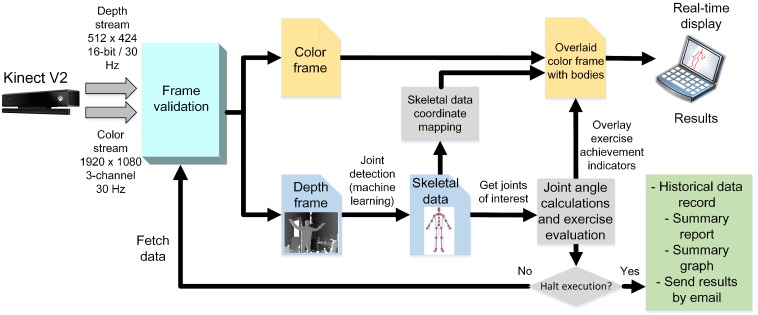
HemoKinect flowchart describing the implemented methodology. The depth frames are processed to obtain the skeletal data from them using a machine learning algorithm, in particular a random decision forest [38]. The skeletal data are then mapped to the color image space using methods coded in Microsoft’s SDK. Then, the overlay image is obtained by drawing the bodies on top of the color image and adding the calculated angles’ achievement indicators. The process continues until the execution is halted. Finally, summary reports and graphs of the exercise are generated.

Then, an overlay color image with skeletal data and performance scores was displayed in real-time on a computer screen. The correspondence between joint coordinates and pixels from the RGB image was obtained with a mapping procedure. The execution can be halted at any moment and reports then automatically generated. The implemented methodology follows the flowchart of Figure 1.

**Figure 2:**
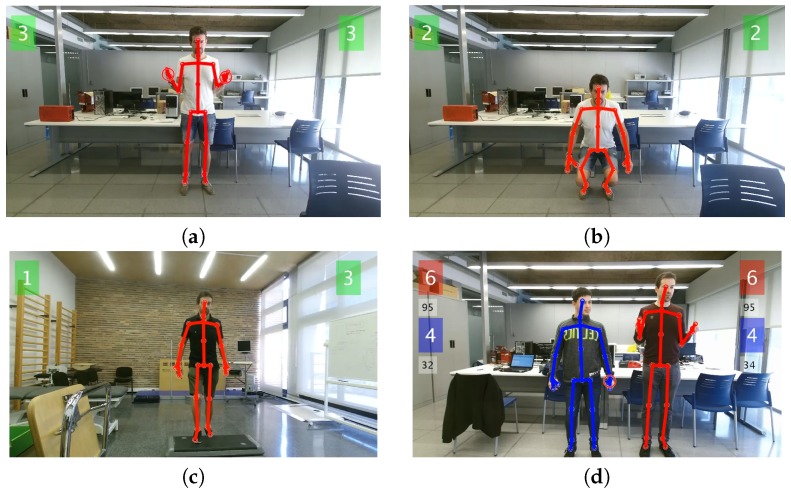
Screenshots of HemoKinect during the different exercises: (**a**) simultaneous elbow flexion exercise, without load; (**b**) knee flexion exercise (squat); (**c**) ankle exercise (step climb); (**d**) elbow flexion exercise in two-player mode. The repetition count for each exercise and individual left/right joint of interest is displayed in the respective left/right corner of the screen. In two-player mode, the repetition count for the second player appears stacked directly below the first players’ count, in a different color. In this last screenshot, the real-time angle measurement of the joints of interest is displayed, as well, in a smaller font, below the respective score box.

The pseudocodes for each exercise: elbow/knee flexion (squat), step climb and balance are summarized in Algorithms 1–3, respectively. The number of accomplished exercises (i.e., the variable flexCount) was displayed on the computer screen for each player, and if desired, the real-time measured angle of the joint of interest could be displayed, as well. Angles were measured by Kinect V2 without any filtering. The only preprocessing done was: (i) discarding the measurement if no bodies were detected or if joints of interest were flagged with the ‘NotTracked’ status and (ii) clipping the 2D joint positions that fell outside the RGB frame. An example screenshot for each exercise is shown in Figure 2, including a sample capture of the two-player mode.

For the balance evaluation, the COM of the player was calculated and a score awarded according to the player’s performance when balancing his or her weight towards different directions. The COM was calculated by distributing the total weight of the patients in body segments using anthropometric data for males [41], as described in Table 1, and assigning the result to the center position of each body segment. The COM was then computed as the weighted average of all body segments’ individual COMs.

**Algorithm 1:** Joint flexion (elbow and knee) count algorithm. The detection is a two-step procedure that firstly checks that a flexed status has been reached, and then, an extension is observed to count the exercise as completed.

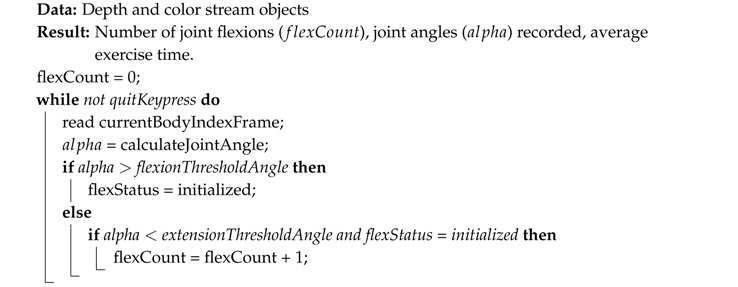



**Algorithm 2:** Step count algorithm. The step detection is based on (a) the general rise of the body, taking the hip center as a reference and (b) the sequential flexion and extension of the knees. The ankle angle history alpha is recorded throughout the duration of the exercise for medical evaluation purposes, but is not used to detect the step climb due to its inaccuracy.

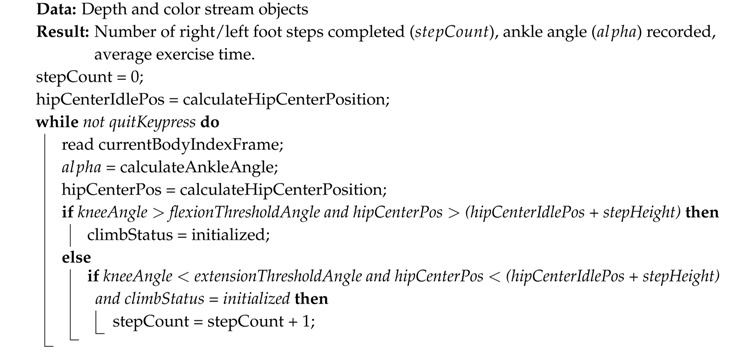



**Algorithm 3:** Balance exercise algorithm. Balance is evaluated in 8 directions in the XZ plane, targeted sequentially in clockwise order: north (N), northeast (NE), east (E), southeast (SE), south (S), southwest (SW), west (W) and northwest (NW), where N corresponds to the player’s front-facing direction. The player must swing back to the starting position (idlePos) before each change of direction. The scores’ array contains the percentages of time spent by the subject in each of the target balance spots.

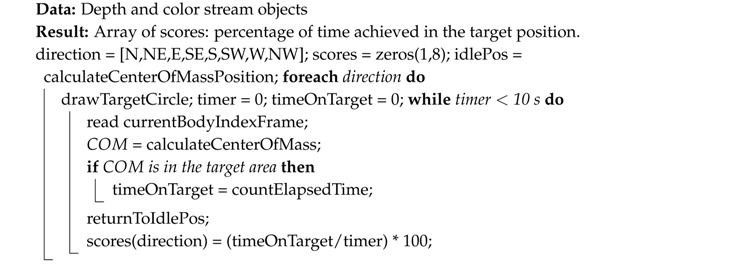



For a segment with start and end joint positions $s_{1}$ and $s_{2}$ ($s_{1},s_{2}\in R^{3}$), respectively, the segment’s COM would be given by Equation (2).

(2) $$s_{COM}=s_{1}+\frac{s_{2}-s_{1}}{2}$$

The COM position $b_{COM}$ of a body with mass *M* is then given by the average of COM positions of N=16 body segments, each one with mass $M_{i}$, i=1,…,N, as shown by Equation (3).

(3) $$b_{COM}=\frac{\sum_{i=1}^{N}M_{i}\cdot s_{COM_{i}}}{M}$$

**Figure 3:**
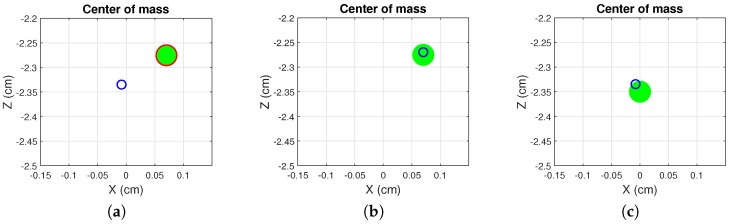
Sample screenshots of HemoKinect’s balance exercise. The player’s COM is represented by a blue circumference. The current target position is represented by a green circle of radius 1.5 cm, whose edge changes color from red to green when the player’s COM is inside it. The figure shows: (**a**) the player has not reached the current target balance position, and the circle’s edge color is red; (**b**) the player reaches the current target balance position (NE), and the edge color changes to green; (**c**) the player reaches the starting position (idlePos) between two successive balance positions.

The target and COM positions are represented by circles in the XZ plane, being Δx the transverse and Δz the longitudinal displacement, respectively. The target circle changes color from red to green when the COM is inside the target area. Sample screenshots of the balance exercise are shown in Figure 3. Three difficulty levels with increasing COM excursion requirements have been implemented. These levels were defined empirically to allow for a mean score for the easiest difficulty level (over all directions) near 50% for the patient with the highest arthropathy and a mean score near 90% for those with no arthropathies. The three levels of difficulty were evaluated sequentially for each patient.

The time granted for the player to reach and stay in the target positions was 10 s in every case. This value was set empirically according to the participants’ feedback and to allow enough time for a reliable score measurement taking into account the different learning curves and reaction times of participants. The outcome of the algorithm is expressed as the percentage of success:

(4) $$score=\frac{Time\;on\;target}{Time\;allowed\;per\;target}$$

where Timeallowedpertarget = 10 s, as mentioned above.

### 2.3. Population

The control population for the study was composed of 10 subjects (age = 35 ± 6 years, height = 172 ± 9 cm, weight = 67 ± 11 kg), and the hemophilic population consisted of 8 subjects (age = 35 ± 10 years, height = 177 ± 6 cm, weight = 89 ± 30 kg). It is worth remarking that the hemophilic population was only composed of males, as hemophilia affects only males, while the control population was composed of both males and females, but this aspect was not significant, as it did not affect Kinect’s joint detection process.

The inclusion criteria for the hemophilic population were: (i) patients must be diagnosed with hemophilia; (ii) over the age of 18; (iii) suffering from arthropathies. With regard to controls, the inclusion criteria were: (i) healthy motor capabilities; (ii) over the age of 18.

Previous approval for this study was obtained from the Ethical Committee of biomedical research of the University and Polytechnic Hospital La Fe (Registry Number 2015/0404). All the procedures were performed in accordance with the principles of the Declaration of Helsinki of the World Medical Association.

## 3. Results

**Figure 4:**
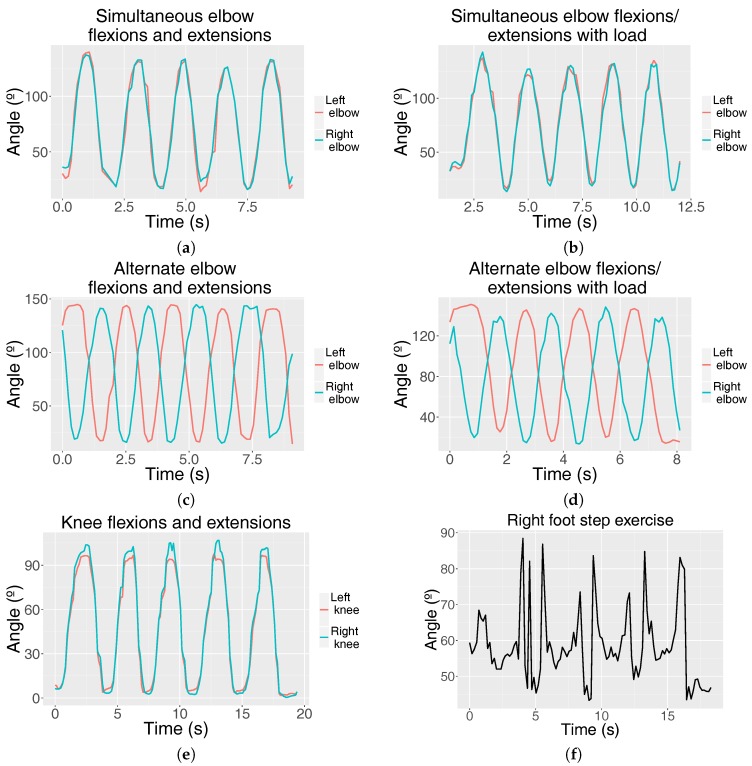
Graphs representing the joint angle (°) fluctuation of different exercises over time (s): simultaneous elbow flexions and extensions (**a**) without load and (**b**) with load; alternate elbow flexions and extensions (**c**) without load and (**d**) with load; (**e**) knee flexions and extensions (squats) and (**f**) right ankle angle fluctuation when performing four right-foot step climbs and descents.

Example plots of each exercise for random patients are shown in Figure 4. For the elbow exercises, the threshold variables *flexionThresholdAngle* and *extensionThresholdAngle* were set to 50${}^{{}^{\circ}}$ and 100${}^{{}^{\circ}}$, respectively. For the knee flexion exercise, the variables *flexionThresholdAngle* and *extensionThresholdAngle* were set to 70${}^{{}^{\circ}}$ and 30${}^{{}^{\circ}}$, respectively. These values were selected according to the doctors’ advice taking into account the mobility limitations of the patients due to arthropathies, while allowing the correct exercise count in controls and avoiding false positive detection (i.e., counting flexions by mistake when walking normally).

Five series of five repetitions per series were collected for each type of exercise and participant. We measured the achievement rate, i.e., the percentage of exercises correctly performed as judged by HemoKinect, for each type of exercise.

For elbow and knee exercises, a 100% of achievement rate was obtained for the control population. In the hemophilic group, the achievement rate for the elbow was also 100%. Table 2 and Table 3 show the rest of the achievement rates, for the control and the hemophilic population, respectively. A perfect performance was measured for the knee exercise for half of the patients, and the average achievement rates over all patients were high (85% for the left knee and 86% for the right knee), except for those who presented high levels of arthropathy in their knees, which did not allow them to reach the detection threshold in some repetitions. As mentioned in Algorithm 3, HemoKinect relies on the knee angle in combination with the hip center position elevation and descent as elements used to detect step climbs and descents. In this manner, step climbs were successfully counted independently on both feet in about 78% of the cases for the right foot step and 75% for the left one in the control population. The patients’ achievement rate falls approximately by 15% with respect to the controls’ rate.

**Figure 5:**
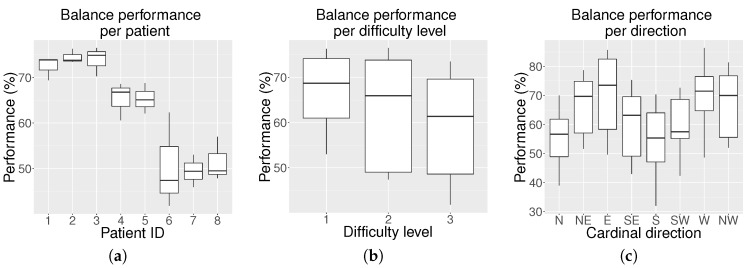
Average balance results (%) on the hemophilic population for all eight cardinal and intermediate positions: (**a**) box plot of average balance performance per patient including all three levels; (**b**) box plot of average balance performance per level for all patients; and (**c**) box plot of the average balance performance per cardinal direction for all patients and levels.

The balance exercise was evaluated using only the hemophilic population. Statistics have been obtained by averaging the score per patient/difficulty level/cardinal direction. The results are shown in the box plots of Figure 5.

Figure 5a shows that each participant obtained a different balance performance. Three patients presented an average performance below 50%, two patients a performance close to 65% and the remaining three patients close to 75%. As expected, Figure 5b shows that the balance performance decreased as the difficulty level increased. Kruskal–Wallis analysis revealed that there were significant differences among the levels of difficulty (*p*-value = 0.013). Multiple comparisons find differences (*p*-value = 0.010) between the performance at Level 1 (median = 68.50 and interquartile range = [58.00–76.00]) and at Level 3 (60.00 [46.25–71.75]. However, no significant differences were found between Level 2 (65.50 [51.00–73.75]) and Level 1 or between Level 2 and Level 3.

The Kruskal-Wallis analysis also revealed that there existed significant differences in the balance performance as a function of the target direction (*p*-value < 0.001). Differences were found between the following directions: S (56.00 [43.75–67.00]) and W (73.50 [65.25–77.00]; *p*-value = 0.008), S and NW (70.00 [57.75–79.75]; *p*-value = 0.042), S and E (72.00 [57.25–85.50]; *p*-value = 0.004), N (56.00 [45.00–67.25]) and E (*p*-value = 0.005), as well as between N and W (*p*-value = 0.011).

## 4. Discussion

The experiments of elbow flexion/extension and knee flexion/extension with empirically tuned threshold angles have been able to successfully detect exercise repetitions for the control and hemophilic population. In the performed tests, an elbow flexion/extension success rate of 100% was achieved by both populations. The squats were successfully performed by all controls and most patients involved in the study. The accuracy of Kinect reveals great potential in the systematization of ROM measurements of multiple patients at the medical consultation. Additionally, by saving a record of the joint data, the specialist could detect potential postural injury risks and help prevent them.

There exists a wide range of commercial MoCap sensors that may be used for clinical applications as, for example: Orbbec Astra, RealSense R200, ZED Stereo Camera, RealSense F200, DUO mini lx, Leap Motion and Kinect V2. Kinect V2 presents a series of advantages over the alternative MoCap sensors: it can be used for full skeletal tracking and track multiple bodies simultaneously, which may allow parallel acquisition of data from several patients; it has a relatively low price; it supports a wide variety of software toolkits (Open-Frameworks, Processing, Unity3D, etc.) and languages (C#, C++, JavaScript, Java, etc.); and it has mature drivers and a well-documented, freely-accessible SDK.

Previous studies that used depth sensors for rehabilitation purposes have mainly used the previous version of Kinect (V1). There are mixed opinions on whether this system should be used for clinical evaluation or not. There are authors that report that angle accuracy is not enough for medical purposes [26] and others that state that they can be acceptable for a rehabilitation tool [14]. Kinect V2 possesses an improved depth sensor and higher resolution, together with the ability to track a larger number of bodies and joints per body. As a result, Kinect V2 becomes a valid alternative for clinical applications, as shown by some authors [28]. In our experiments, Kinect V2 has been able to measure elbow and knee angles when the joints of interest are not occluded by any object. Occlusion has proven to be a limitation since the first Kinect V1 [24] and continues to be a source of error in V2. This means that, in all tests, the patient must be placed facing the sensor and within its recommended range (0.5 m–4.5 m) and under uniform lighting.

The limitation when designing ankle exercises is the inaccuracy in the detection of the ankle position provided by Kinect V2. This was also reported by other authors [28,30]. Although a regular pattern is observed in successive ankle flexions and extensions, the positioning of joints that participate in the ankle movement (particularly the foot segment) is greatly distorted by noise. This fact makes all tested exercises based on the measured ankle angle for counting ankle flexions/extensions unreliable. This limitation was partially bypassed by designing an exercise that implies a step detection. The designed algorithm successfully counts climbs and descents, based on the hip center joint position and knee angle, implicitly forcing the adequate mobility of the ankle in each repetition. In some cases, the occlusion of the ankle joint by the step block may cause errors in the calculation of the knee angle, which may lead to misdetection of some repetitions. Assuming this limitation, the step algorithm can detect both right foot and left foot step climbs independently and has reached average performances between 64% and 78%, depending on the population.

Balance training in exergaming applications has been previously addressed in the literature. The first Kinect V1 has been reported to be effective in accurately characterizing changes in the COM and in flexion-extension movements of the lower limbs during balance training [21]. For hemophilic patients’ rehabilitation, a Nintendo Wii Balance Board has been evaluated [34]. However, the Wii relies on pressure distribution, while Kinect composes a 3D point cloud, and our algorithm can extract the actual COM position.

The balance experiment produces interesting results. As observed from Figure 5a, the balance performance varies in each patient. These differences may be due to their physical condition and level of arthropathy. As expected, according to Figure 5b, the general balance performance decreases with the increasing level of difficulty. However, the results for Levels 1 and 2, as well as for Levels 2 and 3 overlap significantly, and the differences are only obvious between Levels 1 and 3. This implies that the change between levels is not abrupt, but rather progressive. The rapid motor learning of the participants during the exercise execution allowed them to take on Level 3 even if some of them started struggling to reach some positions at Level 1.

For anatomical reasons, swaying to reach the player’s front target positions should be easier than swaying to reach the back ones, and this has been proven by multi-directional reach tests [42,43]. This generally agrees with our results, except for the N direction, as observed in Figure 5c. The explanation for this inconsistent behavior is the fact that this was the first direction the patient was asked to reach. Therefore, their reaction time was higher than for the rest of the directions, and as a consequence, their performance decreased. Predictably, the S target direction obtains the lowest scores, as it is affected by the fear of falling factor [43]. In terms of performance per cardinal direction, the Kruskal–Wallis analysis has proven that statistical differences are only found between directions that are not adjacent.

On the whole, the results of this pilot study are very promising. In the future, the amount of participants of the study will be substantially increased thanks to the distribution of the hardware and software, and statistical tests will be carried out for a long-term measurement campaign of HemoKinect against traditional rehabilitation techniques. HemoKinect is also able to measure the execution time of all exercises, and this could be exploited at advanced rehabilitation stages to encourage patients to speed up their exercises and improve their musculoskeletal health.

### Limitations

In October 2017, it was announced that further development for Kinect V2 will be discontinued. Nevertheless, Microsoft will continue to provide support for the Kinect SDK and is working with Intel to provide an alternative [44]. There are other sensors with body tracking SDKs on the market such as Intel’s RealSense [45], VicoVR [46] or Orbbec Astra [47] that could be evaluated as alternatives to Kinect V2.

Kinect V2 applications already developed, such as HemoKinect, will continue to work, and clients will be able to use them without any issue, as long as they are in possession of Kinect V2 hardware and the SDK v2.0. However, thanks to the modularity of HemoKinect code, it can easily be adapted to new hardware that provides the 3D positions of the joints of interest as the above-mentioned sensors.

## 5. Conclusions

The role of exergaming in modern medical motor training and rehabilitation is overtaking traditional methods, as it allows remote patient supervision by exploiting the advantages of telemedicine. This work has validated the Kinect V2 for hemophilic patients’ exercise routines’ evaluation and tracking for the first time, using a completely newly developed software, HemoKinect.

HemoKinect relies on Microsoft’s Kinect SDK 2.0 to obtain 3D joint positions, which are used for joint angle calculation and COM estimation. These joint coordinates are mapped to the RGB image and streamed in real-time on a computer screen.

HemoKinect is able to successfully count: (a) elbow flexions and extensions; (b) knee flexions and extensions (squats); (c) step climbs and descents; and (d) measure balance performance towards eight different directions at three levels of difficulty. Additionally, a two-player mode is implemented. HemoKinect also allows saving and sharing the results (reports and graphs) via e-mail.

Although Kinect V2 is generally not aimed at medical application purposes where the measurement accuracy is paramount, its accuracy is enough to register hemophilic patients’ exercises and remotely track their progress and achievements to improve their physical fitness, which is a great step forward in telemedicine applied to hemophilia.

Future work will address the comparison of HemoKinect angle measurements for the joints of interest with other systems that are gold standards, such as clinical goniometers. Another possible line of work would be creating and evaluating more complex exercise routines for hemophilic patients.

## Figures and Tables

**Table 1:** Distribution of body mass for males [41].

Body Segment	Percent of Total Body Mass
Head	8.26%
Thorax	20.10%
Abdomen	13.06%
Pelvis	13.66%
Upper Arm	3.25%
Forearm	1.87%
Hand	0.65%
Thigh	10.50%
Leg	4.75%
Foot	1.43%

**Table 2:** Controls step exercise achievement rate (%). Mean ± standard deviation of 5 series of 5 repetitions per exercise.

Control	Right Step	Left Step
1	80 ± 20	72 ± 11
2	88 ± 18	80 ± 14
3	84 ± 16	80 ± 20
4	76 ± 26	84 ± 22
5	80 ± 28	72 ± 11
6	72 ± 11	64 ± 17
7	72 ± 23	68 ± 18
8	80 ± 25	76 ± 22
9	68 ± 22	76 ± 26
10	80 ± 14	84 ± 26
Mean	78 ± 20	75 ± 19

**Table 3:** Patients’ squat and step exercise achievement rate (%). Mean ± standard deviation of 5 series of 5 repetitions per exercise.

Patient	Right Knee	Left Knee	Right Step	Left Step
1	100	100	64 ± 22	56 ± 17
2	92 ± 11	96 ± 9	52 ± 18	60 ± 20
3	100	100	80 ± 20	76 ± 26
4	72 ± 11	76 ± 9	68 ± 22	64 ± 29
5	100	100	64 ± 22	56 ± 26
6	60 ± 14	52 ± 11	52 ± 18	56 ± 17
7	64 ± 9	60 ± 14	68 ± 11	72 ± 11
8	100	100	80 ± 28	76 ± 17
Mean	86 ± 5	85 ± 5	66 ± 20	64 ± 20
